# Precise QCD predictions for W-boson production in association with a charm jet

**DOI:** 10.1140/epjc/s10052-024-12715-8

**Published:** 2024-04-04

**Authors:** A. Gehrmann-De Ridder, T. Gehrmann, E. W. N. Glover, A. Huss, A. Rodriguez Garcia, G. Stagnitto

**Affiliations:** 1 Institute for Theoretical Physics, ETH, 8093 Zurich, Switzerland; 2 Department of Physics, University of Zürich, 8057 Zurich, Switzerland; 3 Institute for Particle Physics Phenomenology, Durham University, Durham, DH1 3LE UK; 4 Department of Physics, Durham University, Durham, DH1 3LE UK; 5 Theoretical Physics Department, CERN, 1211 Geneva 23, Switzerland; 6 Università degli Studi di Milano-Bicocca and INFN, Piazza della Scienza 3, 20126 Milan, Italy

## Abstract

The production of a *W*-boson with a charm quark jet provides a highly sensitive probe of the strange quark distribution in the proton. Employing a novel flavour dressing procedure to define charm quark jets, we compute *W*+charm-jet production up to next-to-next-to-leading order (NNLO) in QCD. We study the perturbative stability of production cross sections with same-sign and opposite-sign charge combinations for the *W* boson and the charm jet. A detailed breakdown according to different partonic initial states allows us to identify particularly suitable observables for the study of the quark parton distributions of different flavours.

## Introduction

The quark and gluon content of the proton is described by parton distributions functions (PDFs), which parametrise the probabilities for a given parton species to carry a specific fraction of the longitudinal momentum of a fastly moving proton. PDFs can not be computed from first principles in perturbative QCD, which determines only their evolution with the resolution scale [1, 2]. The initial distributions for all quark and antiquark flavours and gluons are thus determined from global fits [3–7] to a large variety of experimental data from high-energy collider and fixed-target experiments. The resulting PDFs do not have uniform uncertainties across the different quark flavours, since only some flavour combinations are tightly constrained by precision data, e.g. from inclusive neutral-current structure functions or from vector boson production cross sections. In particular the strange quark and antiquark distributions are mainly constrained from fixed-target neutrino-nucleon scattering data [8, 9].

The production of a massive gauge boson in association with a flavour-identified jet offers a unique possibility to study PDFs for specific quark flavours. *W*+charm-jet production [10–14] is of particular relevance, since its Born-level production cross section is largely dominated by initial states colliding a gluon and a strange quark. By selecting the *W* charge, strange and anti-strange distributions can be probed separately. The production of *W* bosons with heavy quarks has been studied by ATLAS [15], CMS [16–18] and LHCb [19]. However, these measurements use various different prescriptions to identify the presence of the heavy flavour, such as for example by tagging a specific heavy hadron species, or by a flavour-tracking in the jet clustering.

The definition and identification of jet flavour [20] is highly non-trivial due to possible issues with infrared and collinear safety (IRC) related to the production of secondary quark-antiquark pairs that can partially or fully contribute to the jet flavour. Several proposals to assign flavour to jets in an IRC safe were recently put forward [21–24], and a generic prescription to test the IRC safety of jet flavour definitions has been formulated [24].

To include precision data from *W*+charm production processes in global PDF fits, higher-order QCD corrections to the respective production cross sections are required. These have been computed previously for *W*+charm-jet production to next-to-next-to-leading order (NNLO) [13, 14], while *W*+charm-hadron production is currently only known to next-to-leading order (NLO) by combining the identified quark production at this order with a parton-shower and hadronization model [25, 26].

In this paper, we present a new NNLO computation of *W*+charm-jet production, employing the flavour dressing procedure [23] to define charm quark jets. Compared to other prescriptions for the flavour assignment of jets, the flavour dressing procedure has the advantage of retaining the exact kinematics of the anti-$k_T$ jet algorithm. Our calculation is performed in the NNLOJET parton-level event generator framework [27], which implements the antenna subtraction method [28–30] for the handling of infrared singular real radiation configurations up to NNLO. Using this new implementation, we investigate the effects of higher-order QCD corrections on different charge-identified *W*+charm-jet cross sections and kinematical distributions. We decompose the predictions according to the partonic composition of the initial state, which allows us to quantify the sensitivity of different types of observables on the PDFs of strange quarks and of other quark flavours.

The paper is structured as follows. In Sect. 2, we describe the calculation of the NNLO QCD corrections, elaborating in particular on the extensions to antenna subtraction and to the NNLOJET code required for flavour and charge tracking. Section 3 describes the results for the flavour and charge identified distributions at NNLO and investigates their perturbative stability. We perform a detailed decomposition into partonic channels in Sect. 4 and discuss various observations that can be made based on this channel breakdown. We conclude with a summary in Sect. 5.

## Details of the calculation

Our calculation of the NNLO corrections to $W+c$-jet production is based on the NNLOJET parton-level event generator framework, which implements the antenna subtraction method [28–30] for the cancellation of infrared singular terms between real radiation and virtual contributions. It builds upon the NNLOJET implementation of $W+$jet production [31, 32]. The NNLO corrections consist of three types of contributions: two-loop virtual (double virtual, VV), single real radiation at one loop (real-virtual, RV) and double real radiation (RR). The matrix elements for these contributions to $W+$jet production are well-known and can be expressed in compact analytic form [33–39].

The $W+$jet implementation in NNLOJET had to be extended in various aspects to enable predictions for jets containing an identified charm quark, as described in detail in the following subsections. The full dependence of the subprocess matrix elements on the initial- and final-state quark flavours (including CKM mixing effects) had to be specified, a flavour dressing procedure for the assignment of jet flavour [23] and the flavour tracking in all stages of the calculation had to be implemented, and the antenna subtraction terms had to be adapted to allow for full flavour and charge tracking.

### Implementation of CKM flavour mixing

Quark flavour mixing effects in processes involving final-state $W^\pm$ bosons were previously included in NNLOJET by constructing CKM-weighted combinations of incoming parton luminosities. This prescription allowed to minimise the number of evaluations of subprocess matrix elements and associated subtraction terms per phase space point, thereby contributing to the numerical efficiency of the calculation. This implementation relies on a flavour-agnostic summation over all final-state quarks and antiquarks, and does not allow to assign a specific quark flavour to any final state object.

In the case of $Z+b$ production [40] and $Z+c$ production [41], the respective final-state quark flavours could be extracted, starting from the *Z*+jet matrix elements, in a rather straightforward manner by excluding them from the flavour sum, and keeping the identified flavour contribution as a separate process. For $W+c$ production, flavour identification required to dress all matrix elements with the respective CKM factors at the *W* interaction vertex, thereby fixing the associated quark flavours in the initial and final state. Where appropriate, initial state flavour combinations were again concatenated into weighted combinations of parton luminosities for computational efficiency, while final-state flavours (and quark charges) were clearly identified for all subprocesses. The numerically negligible contributions involving $|V_{cb}|$ are omitted throughout.

### Flavour dressing of jets and flavour tracking in NNLOJET

In order to compute observables sensitive to the flavour of the particles involved, it is necessary to retain the flavour information in both matrix elements and subtraction terms. A mechanism of flavour tracking has been implemented in NNLOJET, see [42] for an overview of this procedure. Here we stress the fact that the reduced matrix elements within the same subtraction term can have different flavour structures, because they are related to different unresolved limits of the matrix element. This observation will be crucial in Sect. 2.3 below.

Once we have the flavour information of final-state particles at our disposal, it is important to adopt an infrared and collinear (IRC) safe definition of flavour of hadronic jets. In other words, we require that the flavour of jets is not affected by the emission of soft particles and/or collinear splittings (e.g. $g \rightarrow c\bar{c}$), in order to guarantee the local cancellation of singularities between matrix elements and subtraction terms. Several proposals to assign flavour to jets in an IRC safe way have recently appeared [21–24]. In the present analysis, we will adopt the flavour dressing algorithm of [23]. The key property of this approach is that the flavour assignment of jets is entirely factorised from the initial jet reconstruction. Hence, we can define the flavour of anti-$k_t$ jets – the de facto standard at the LHC – in an IRC safe way.

However, in Ref. [24] it has been shown that the original formulation of the flavour dressing algorithm as presented in [23] starts being IRC unsafe at higher orders. This has been proven by looking at explicit partonic configurations with many hard and soft/collinear particles and by developing a dedicated numerical framework for fixed-order tests of IRC safety.

After the findings of [24], the flavour dressing algorithm has been adjusted, and the new version passes the numerical fixed-order tests of [24] up to $\mathcal {O}(\alpha _s^6)$. In the new formulation, flavoured *clusters* are no longer used; instead, all particles directly enter the flavour assignment step, and we run a sequential recombination algorithm by considering both distances between particles and between particles and jets.

### Charge tracking in quark-antiquark antenna functions

Previous NNLOJET calculations of $Z+b$ production [40] and $Z+c$ production [41] always summed over the charges of the identified quarks, i.e. $q=(b,c)$ could be either a flavour-identified quark or a flavour-identified antiquark. Furthermore, in any given subprocess, quarks and antiquarks of the same flavour always come in pairs in these calculations. In the current calculation of $W+c$-jet production in NNLOJET, this is no longer the case, since a charm quark that has a direct coupling to the *W*-boson will be associated with its corresponding isospin partner (predominantly the strange antiquark $\bar{s}$, or the CKM-suppressed down-antiquark $\bar{d}$). Moreover, it is desirable to be able to distinguish charm quarks and antiquarks, thereby allowing the study of charge correlations between the produced *W* boson and the identified charm (anti-)quark (same-sign, SS, and opposite-sign, OS, observables), as is done in the experimental analyses.

**Table 1:** Inclusive and exclusive fiducial cross sections for $\sigma (W^+ + c\text {-jet})$ in OS−SS and OS+SS cases. We show the Monte Carlo errors as an uncertainty on the last digit while the percentage errors show the 7-point scale variation envelope

$W^+ + c\text {-jet}$	OS−SS incl.	OS−SS excl.	OS+SS incl.	OS+SS excl.
$\sigma ^{\textrm{LO}}$	$91.34(1)^{+ 11.7 \% }_{- 9.5 \% }$	$91.34(1)^{+ 11.7 \% }_{- 9.5 \% }$	$91.34(1)^{+ 11.7 \% }_{- 9.5 \% }$	$91.34(1)^{+ 11.7 \% }_{- 9.5 \% }$
$\Delta \sigma ^{\textrm{NLO}}$	30.46(4)	30.29(4)	39.23(4)	38.13(4)
$\sigma ^{\textrm{NLO}}$	$121.80(4)^{ + 5.6 \% } _{ - 5.4 \% }$	$121.59(4)^{ + 5.6 \% } _{ - 5.4 \% }$	$130.56(4)^{ + 6.9 \% } _{ - 6.3 \% }$	$129.47(4)^{ + 6.8 \% } _{ - 6.2 \% }$
$\Delta \sigma ^{\textrm{NNLO}}$	$-2.3(8)$	$-2.6(7)$	4.5(7)	3.2(7)
$\sigma ^{\textrm{NNLO}}$	$119.5(8)^{ + 0.4 \% } _{ - 1.8 \% }$	$119.0(7)^{ + 0.1\% } _{ - 1.6\% }$	$135.1(8)^{ + 1.2 \% } _{ - 1.9 \% }$	$132.7(7)^{ + 0.6 \% } _{ - 1.5 \% }$

**Table 2:** Inclusive and exclusive fiducial cross sections for $\sigma (W^- + c\text {-jet})$ in OS−SS and OS+SS cases. As in Table 1, we show the Monte Carlo errors as an uncertainty on the last digit while the percentage errors show the 7-point scale variation envelope

$W^- + c\text {-jet}$	OS−SS incl.	OS−SS excl.	OS+SS incl.	OS+SS excl.
$\sigma ^{\textrm{LO}}$	95.782(4)$^{+ 11.7 \% }_{- 9.5 \% }$	95.782(4)$^{+ 11.7 \% }_{- 9.5 \% }$	95.782(4)$^{+ 11.7 \% }_{- 9.5 \% }$	95.782(4)$^{+ 11.7 \% }_{- 9.5 \% }$
$\Delta \sigma ^{\textrm{NLO}}$	32.244(8)	32.004(8)	39.011(8)	38.043(8)
$\sigma ^{\textrm{NLO}}$	128.026(9)$^{+ 5.7 \% }_{- 5.5 \% }$	127.786(9)$^{+ 5.7 \% }_{- 5.5 \% }$	134.794(9)$^{ + 6.6 \% } _{ - 6.1 \% }$	133.826(9)$^{ + 6.5 \% } _{ - 6.0 \% }$
$\Delta \sigma ^{\textrm{NNLO}}$	2.9(5)	2.5(5)	8.2(5)	7.1(5)
$\sigma ^{\textrm{NNLO}}$	130.9(5)$^{+ 0.9 \% }_{- 1.5 \% }$	130.3(5)$^{+ 0.9 \% }_{- 1.5 \% }$	143.0(5)$^{ + 1.5 \% } _{ - 2.5 \% }$	141.0(5)$^{ + 1.1 \% } _{ - 2.4 \% }$

**Table 3:** Inclusive and exclusive fiducial cross sections for the ratio $\mathcal {R}^\pm _c = \sigma (W^+ + c\text {-jet})/\sigma (W^- + c\text {-jet})$ in OS−SS and OS+SS cases. As in Table 1, we show the Monte Carlo errors as an uncertainty on the last digit while the percentage errors show the 31-point scale variation envelope

$\mathcal {R}^{\pm }_{c}$	OS−SS incl.	OS−SS excl.	OS+SS incl.	OS+SS excl.
LO	0.9536(3)$^{+ 21.4 \% }_{- 17.6\% }$	0.9536(3)$^{+ 21.4 \% }_{- 17.6\% }$	0.9536(3)$^{+ 21.4 \% }_{- 17.6\% }$	0.9536(3)$^{+ 21.4 \% }_{- 17.6\% }$
NLO	0.951 (1)$^{ + 8.6 \% } _{ - 8.0 \% }$	0.952(1)$^{ + 8.5 \% } _{ - 8.0 \% }$	0.969(1)$^{ + 10.3 \% } _{ - 9.2 \% }$	0.967(1)$^{ + 10.1 \% } _{ - 9.1 \% }$
NNLO	0.91(1)$^{ + 1.5 \% } _{ - 2.1 \% }$	0.91(1)$^{ + 1.6 \% } _{ - 1.6 \% }$	0.94(1)$^{ + 2.5 \% } _{ - 2.9 \% }$	0.94(1)$^{ + 2.8 \% } _{ - 2.6 \% }$

This charge identification requires a slight extension of the antenna subtraction formalism to accommodate the charge-tracking in the quark-antiquark antenna functions. The requirement of charge-tracking can be illustrated with an example. We consider the gluon-induced double real radiation contribution to $W^- c$ production:

1 $$\begin{aligned} g(p_1)g(p_2)\rightarrow W^-(q) c(p_i){\bar{s}}(p_j) g(p_k), \end{aligned}$$

which contains the colour-ordered subprocess matrix element:

2 $$\begin{aligned} \tilde{B}_{3}^0(i_c, 1_g,k_g,2_{\tilde{g}},j_{\bar{s}}), \end{aligned}$$

at first subleading colour level. The full colour decomposition of the two-quark, three gluon matrix elements is described for neutral gauge bosons in Section 4.2 of [43], and $\tilde{B}_{3,W^-}^0$ is obtained from the $\tilde{A}_5^0$ defined there by replacing the neutral gauge boson by a $W^-$. Here $\tilde{g}$ denotes the abelian-like gluon that is colour-connected only to the quark-antiquark pair, while the other two gluons are colour-connected to each other and to either the quark or the antiquark. The partonic labelling of the momenta is in all-final kinematics, with incoming particles denoted by momenta 1 and 2.

The subtraction of triple-collinear limits corresponding to the splitting of the incoming (non-abelian) gluon into a quark-antiquark-gluon cluster (from which either the quark or the antiquark enters the hard subprocess) requires the leading-colour quark-antiquark antenna function $A_4^0(i_q,1_g,k_g,j_{\bar{q}})$. This antenna function contains two triple collinear limits: TC($q_i\parallel g_1 \parallel g_k$) and TC($\bar{q}_j\parallel g_k \parallel g_1$). The associated triple-collinear splitting functions correspond to different colour orderings and are not identical. In these two limits, (2) factorises as follows onto the tree-level quark-antiquark-gluon matrix element $B_{1,W^-}^0$:

3 $$\begin{aligned} \tilde{B}_{3}^0(i_c, 1_g,k_g,2_{\tilde{g}},j_{\bar{s}})&{\mathop {\longrightarrow }\limits ^{i\parallel 1 \parallel k}}&P_{q_i\parallel g_1 \parallel g_k} B_{1}^0(\hat{1}_c,2_g,j_{\bar{s}}) , \nonumber \\ \tilde{B}_{3}^0(i_c, 1_g,k_g,2_{\tilde{g}},j_{\bar{s}})&{\mathop {\longrightarrow }\limits ^{j\parallel k \parallel 1}}&P_{ \bar{q}_j\parallel g_k \parallel g_1} B_{1}^0(i_c,2_g,\hat{1}_{\bar{s}}) , \end{aligned}$$

where $\hat{1}$ denotes the composite momentum that flows into the hard matrix element after the collinear splitting. It becomes evident that only the $\bar{q}_j\parallel g_k \parallel g_1$ limit factorises onto a matrix element corresponding to a $W^-c$ final state, while the $q_i\parallel g_1 \parallel g_k$ leads to a $W^- \bar{s}$ final state with an anti-charm quark in the initial state of the reduced matrix element. To construct the RR subtraction term for (2), one must therefore split $A_4^0(i_q,1_g,k_g,j_{\bar{q}})$ into sub-antenna functions that contain only a well-defined subset of its infrared limits. The split is analogous to the split that is used for the initial-final quark-antiquark antenna function at NLO [28, 29]:

4 $$\begin{aligned} A_3^0(i_q,1_g,j_{\bar{q}}) = a_3^0(i_q,1_g,j_{\bar{q}}) + a_3^0(j_{\bar{q}},1_g,i_q), \end{aligned}$$

where $a_3^0(i_q,1_g,j_{\bar{q}})$ contains only the $q_i\parallel g_1$ collinear limit.

The decomposition into sub-antennae reads as follows:

5 $$\begin{aligned}{} & {} A_4^0(i_q,1_g,k_g,j_{\bar{q}}) \nonumber \\{} & {} \quad = a_4^{0,c}(i_q,1_g,k_g,j_{\bar{q}}) + a_4^{0,d}(i_q,1_g,k_g,j_{\bar{q}}), \end{aligned}$$

where we require $a_4^{0,c}$ to contain all limits where the incoming gluon $1_g$ becomes collinear to quark $i_q$ and $a_4^{0,d}$ to contain all limits where it becomes collinear to antiquark $j_{\bar{q}}$. Consequently, these sub-antenna functions should contain the following double unresolved (triple collinear, TC, double single collinear, DC, and soft-collinear, SC) limits:

$$\begin{aligned} a_4^{0,c}(i_q,1_g,k_g,j_{\bar{q}})&\supset&\textrm{TC}(q_i\parallel g_1 \parallel g_k), \\{} & {} \textrm{DC}(q_i\parallel g_1, \bar{q}_j\parallel g_k), \\{} & {} \textrm{SC}(q_i\parallel g_1,g_k\hbox { soft}), \\ a_4^{0,d}(i_q,1_g,k_g,j_{\bar{q}})&\supset&\textrm{TC}(\bar{q}_j\parallel g_k \parallel g_1), \\{} & {} \textrm{SC}(\bar{q}_j\parallel g_1,g_k\hbox { soft}) . \end{aligned}$$

The behaviour in the single unresolved limits is more complicated, since the sub-antenna functions should factor onto appropriate three-parton antenna functions $A_3^0$ or their respective sub-antennae:

6 $$\begin{aligned} a_4^{0,c}(i_q,1_g,k_g,j_{\bar{q}})&{\mathop {\longrightarrow }\limits ^{i\parallel 1}}&P_{q_i\parallel g_1} A_3^0(\hat{1}_q,k_g,j_{\bar{q}}), \nonumber \\ a_4^{0,d}(i_q,1_g,k_g,j_{\bar{q}})&{\mathop {\longrightarrow }\limits ^{i\parallel 1}}&0 ,\nonumber \\ a_4^{0,c}(i_q,1_g,k_g,j_{\bar{q}})&{\mathop {\longrightarrow }\limits ^{k\parallel 1}}&P_{g_k\parallel g_1} a_3^0(i_q,\hat{1}_g,j_{\bar{q}}) , \nonumber \\ a_4^{0,d}(i_q,1_g,k_g,j_{\bar{q}})&{\mathop {\longrightarrow }\limits ^{k\parallel 1}}&P_{g_k\parallel g_1} a_3^0(j_{\bar{q}},\hat{1}_g,i_q) , \nonumber \\ a_4^{0,c}(i_q,1_g,k_g,j_{\bar{q}})&{\mathop {\longrightarrow }\limits ^{k\parallel j}}&P_{q_j\parallel g_k} a_3^0(i_q,1_g,(jk)_{\bar{q}}) , \nonumber \\ a_4^{0,d}(i_q,1_g,k_g,j_{\bar{q}})&{\mathop {\longrightarrow }\limits ^{k\parallel j}}&P_{q_j\parallel g_k} a_3^0(j_{\bar{q}},1_g,(jk)_q) , \nonumber \\ a_4^{0,c}(i_q,1_g,k_g,j_{\bar{q}})&{\mathop {\longrightarrow }\limits ^{k\hbox { soft}}}&S_{1kj} a_3^0(i_q,1_g,i_{\bar{q}}), \nonumber \\ a_4^{0,d}(i_q,1_g,k_g,j_{\bar{q}})&{\mathop {\longrightarrow }\limits ^{k\hbox { soft}}}&S_{1kj} a_3^0(j_{\bar{q}},1_g,i_q), \end{aligned}$$

where (*jk*) denotes the momentum of the collinear final-state cluster, *P* are the collinear splitting factors and *S* are eikonal factors.

The decomposition (5) of $A_4^0(i_q,1_g,k_g,j_{\bar{q}})$ into its sub-antennae starts from its triple collinear behaviour. The triple collinear limit TC($q_i\parallel g_1 \parallel g_k$) is characterised by the Mandelstam invariants $(s_{i1k},s_{i1},s_{1k},s_{ik})$ becoming simultaneously small, while the TC($\bar{q}_j\parallel g_k \parallel g_1$) corresponds to $(s_{1kj},s_{1k},s_{kj},s_{1j})$ becoming small. From these sets, $s_{ik}$ and $s_{1j}$ do not appear as denominators in $A_4^0(i_q,1_g,k_g,j_{\bar{q}})$ due to its colour-ordering. Any denominator containing $s_{i1k}$ or $s_{i1}$ is then partial fractioned against any denominator with $s_{1kj}$ or $s_{1k}$, using e.g.

7 $$\begin{aligned} \frac{1}{s_{i1k}s_{1kj}} = \frac{1}{s_{i1k}(s_{i1k}+s_{1kj})}+ \frac{1}{s_{1kj}(s_{i1k}+s_{1kj})}\,, \end{aligned}$$

followed by a power-counting to assign terms that are sufficiently singular (two small invariants) in TC($q_i\parallel g_1 \parallel g_k$) to $a_4^{0,c}(i_q,1_g,k_g,j_{\bar{q}})$ and terms from TC($\bar{q}_j\parallel g_k \parallel g_1$) to $a_4^{0,d}(i_q,1_g,k_g,j_{\bar{q}})$. Terms that contribute in both limits (i.e. those ones that contain $s_{1k}$ in the denominator) remain unassigned at this stage. This procedure already ensures the correct assignment of $\textrm{DC}(q_i\parallel g_1, \bar{q}_j\parallel g_k)$ and $\textrm{C}(q_i\parallel g_1)$ to $a_4^{0,c}(i_q,1_g,k_g,j_{\bar{q}})$.

In a second step, the simple collinear limits $\textrm{C}(\bar{q}_j\parallel g_k)$ and $\textrm{C}({g}_1\parallel g_k)$ as well as the soft limit $\textrm{S}(k)$ are analysed by marking the respective progenitor terms in $A_4^0(i_q,1_g,k_g,j_{\bar{q}})$ and assigning them to either $a_4^{0,c}$ or $a_4^{0,d}$ (taking account of single unresolved behaviour of the previously assigned triple-collinear terms), such that (6) are fulfilled. For simplicity, the limits are taken in all-final kinematics, but the resulting decompositions are valid in any kinematics. The limits $\textrm{C}(\bar{q}_j\parallel g_k)$ and $\textrm{S}(k)$ are straightforward, while $\textrm{C}({g}_1\parallel g_k)$ is more involved due to the occurrence of angular terms in the gluon-to-gluon splitting. In the implementation of the antenna subtraction method, these terms are removed from matrix elements and subtraction terms by appropriate averages over phase space points that are related by angular rotations. The decomposition into $a_4^{0,c}$ or $a_4^{0,d}$ must ensure that these averages still work at the level of the sub-antenna functions.

The limit $\textrm{C}({g}_1\parallel g_k)$ is taken using a Sudakov parametrization of the momenta [44]:

8 $$\begin{aligned} p_1^\mu= & {} z p^\mu + k_T^\mu - \frac{k_T^2}{z} \frac{1}{2p\cdot n} n^\mu , \nonumber \\ p_k^\mu= & {} (1-z) p^\mu - k_T^\mu - \frac{k_T^2}{1-z} \frac{1}{2p\cdot n} n^\mu \end{aligned}$$

with

9 $$\begin{aligned} 2p_1\cdot p_k= & {} -\frac{k_T^2}{z(1-z)}, \quad p^2 = 0, \nonumber \\ n^2= & {} 0, \quad p\cdot k_T = 0, \quad n\cdot k_T = 0. \end{aligned}$$

In this parametrization, $p^\mu$ is the composite momentum of the collinear cluster, while $n^\mu$ is an arbitrary light-like direction. The collinear limit is then taken as Taylor expansion in $k_T^\mu$, retaining terms up to second power, and performing the angular average in $d=4-2\epsilon$ dimensions over the transverse direction of $k_T^\mu$ in the (*p*, *n*) center-of-momentum frame:

10 $$\begin{aligned} \langle k_T^\mu \rangle = 0, \quad \langle k_T^\mu k_T^\nu \rangle = \frac{k_T^2}{d-2} \left( g^{\mu \nu } - \frac{p^\mu n^\nu +p^\nu n^\mu }{p\cdot n}\right) .\nonumber \\ \end{aligned}$$

The reference momentum $n^\mu$ is kept symbolic. The collinear $\textrm{C}({g}_1\parallel g_k)$ behaviour of the full antenna function $A_4^0(i_q,1_g,k_g,j_{\bar{q}})$ is independent on $n^\mu$, but individual terms extracted from it will display a dependence on $n^\mu$ in the collinear limit. The terms are sorted into $a_4^{0,c}$ or $a_4^{0,d}$ in such a manner that both sub-antenna functions remain independent on $n^\mu$ when taking the collinear limit.

The decomposition into sub-antennae introduces polynomial denominators in the invariants into $a_4^{0,c}$ and $a_4^{0,d}$. These are unproblematic at the level of the unintegrated subtraction terms, but may pose an obstruction to their analytical integration. However, when summing over all colour orderings and by allowing for momentum relabelling of different phase-space mappings that correspond to the same phase-space factorization (retaining of course the correct identification of the identified charm quark in the reduced matrix element), we can always combine $a_4^{0,c}$ and $a_4^{0,d}$ into a full $A_4^0$ at the level of the integrated subtraction term at VV level. Consequently, no new integrated antenna functions are needed.

The subleading-colour $\tilde{A}_4^0(i_q,1_g,k_g,j_{\bar{q}})$ antenna function and the $B_4^0(i_q,1_{q'},k_{\bar{q}'},j_{\bar{q}})$ antenna function containing a secondary quark-antiquark pair were decomposed in the same way. In addition, the quark-antiquark one-loop antenna functions present at real-virtual level and given in the final-final kinematics in [28] also need to be decomposed into sub-antennae. The decomposition is however much easier than for the four-parton antennae, as those capture only single unresolved limits of the real-virtual matrix-elements.

## Results

### Numerical setup

We consider a generic setup for Run 2 at $\sqrt{s}=13$ TeV. In particular, the following fiducial cuts for jets and charged leptons are applied:

$$\begin{aligned}{} & {} p_{T,\ell }> 27~\textrm{GeV},\quad |y_{\ell }|< 2.5,\\{} & {} p_{T,j}> 20~\textrm{GeV},\quad |\eta _{j}| < 2.5, \Delta R(j,\ell )> 0.4,\\{} & {} E_{T,\textrm{miss}}> 20~\textrm{GeV},\quad M_{T,W} > 45~\textrm{GeV}. \end{aligned}$$

The transverse mass of the *W*-boson is defined as

11 $$\begin{aligned} M_{T,W} = \sqrt{2\,p_{T,\ell }\,E_{T,\textrm{miss}}\,(1-\cos \Delta \phi _{\ell \nu })}. \end{aligned}$$

The jets are reconstructed with the anti-$k_T$ algorithm [45] with $R=0.4$. The selection of *c*-jets is performed using the flavour dressing procedure described in [23].

**Fig. 1:**
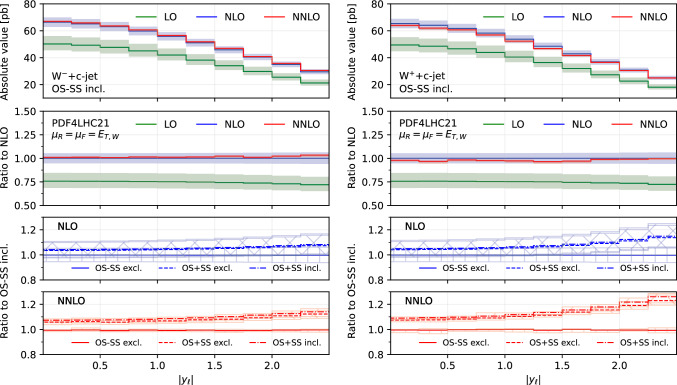
Comparison of predictions for the absolute rapidity of the lepton $|y_{\ell }|$, in the $W^-$+$c$-jet (left) and $W^+$+$c$-jet (right) processes. Panels from top to bottom: differential distribution at different orders; ratio of differential distributions to NLO; ratio of (OS−SS, excl.), (OS+SS, excl.) and (OS+SS, incl.) distributions to (OS−SS, incl.) at NLO; same for NNLO

**Fig. 2:**
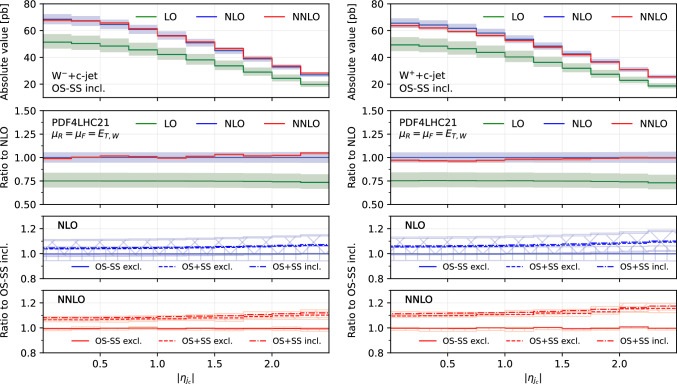
Comparison of predictions for the absolute pseudorapidity of the leading $c$-jet $|\eta _{j_c}|$, in the $W^-$+$c$-jet (left) and $W^+$+$c$-jet (right) processes. Panels from top to bottom: differential distribution at different orders; ratio of differential distributions to NLO; ratio of (OS−SS, excl.), (OS+SS, excl.) and (OS+SS, incl.) distributions to (OS−SS, incl.) at NLO; same for NNLO

We use the PDF4LHC21 Monte Carlo PDF set [46], with $\alpha _s(M_Z) = 0.118$ and $n_f^{\textrm{max}} = 5$, where both the PDF and $\alpha _s$ values are accessed via LHAPDF [47]. For the electroweak input parameters, the results are obtained in the $G_{\mu }$-scheme, using a complex mass scheme for the unstable internal particles, and we adopt the following values for the input parameters:

$$\begin{aligned} M_{Z}^\textrm{os}= & {} 91.1876~\textrm{GeV}, \quad \Gamma _{Z}^\textrm{os} = 2.4952~\textrm{GeV},\\ M_{W}^\textrm{os}= & {} 80.379~\textrm{GeV},\quad \Gamma _{W}^\textrm{os} = 2.085~\textrm{GeV},\\ G_\mu= & {} 1.1663787 \times 10^{-5}~\textrm{GeV}^{-2}. \end{aligned}$$

We further adopt a non-diagonal CKM matrix, thus allowing for all possible charged-current interactions with massless quarks, with Wolfenstein parameters $\lambda = 0.2265$, $A = 0.79$, ${\bar{\rho }} = 0.141$ and ${\bar{\eta }} = 0.357$ [48].

For differential distributions, the impact of missing higher-order corrections is assessed using the conventional 7-point scale variation prescription: the values of factorisation ($\mu _F$) and renormalisation ($\mu _R$) scales are varied independently by a factor of two around the central scale $\mu _0 \equiv E_{T,W}$, with the additional constraint that $\frac{1}{2} \le \mu _F/\mu _R \le 2$. The transverse energy $E_{T,W}$ is defined as

12 $$\begin{aligned} E_{T,W} = \sqrt{ M_{\ell \nu }^2 + p_{T,\ell \nu }^2 }, \end{aligned}$$

with $M_{\ell \nu }$ the invariant mass of the lepton-neutrino pair, and $p_{T,\ell \nu } \equiv |\vec {p}_{T,\ell \nu }|$ the transverse momentum of the lepton-neutrino system.

When considering theoretical predictions for the ratio of distributions, we estimate the uncertainties in an uncorrelated way between the numerator and denominator i.e. by considering

13 $$\begin{aligned} R (\mu _R,\mu _F;\mu '_R,\mu '_F) = \frac{\sigma ^{W^{+}+c\text {-jet}}(\mu _R,\mu _F)}{\sigma ^{W^{-}+c\text {-jet}}(\mu '_R,\mu '_F)}, \end{aligned}$$

providing a total of 31-points when dropping the extreme variations in any pair of scales.

Our default setup requires each event to have at least one *c*-jet (*inclusive* setup). We further apply the OS−SS subtraction: we separately consider events where the lepton from the *W*-decay has the opposite sign (OS) or the same sign (SS) of that of the $c$-jet, and then we take the difference of the corresponding distributions (OS−SS). In our fixed-order predictions, the sign of the $c$-jet is defined as the net sign of all the flavoured particles (i.e. *c*-quarks) that are assigned to the jet at the end of the flavour dressing procedure. When more than one *c*-jet is present, the leading-$p_T$ *c*-jet is used to define the OS−SS subtraction.

**Fig. 3:**
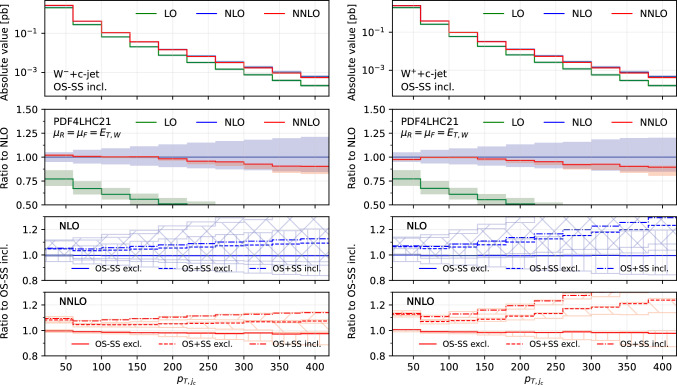
Comparison of predictions for the transverse momentum of the leading $c$-jet $p_{T,j_c}$, in the $W^-$+$c$-jet (left) and $W^+$+$c$-jet (right) processes. Panels from top to bottom: differential distribution at different orders; ratio of differential distributions to NLO; ratio of (OS−SS, excl.), (OS+SS, excl.) and (OS+SS, incl.) distributions to (OS−SS, incl.) at NLO; same for NNLO

**Fig. 4:**
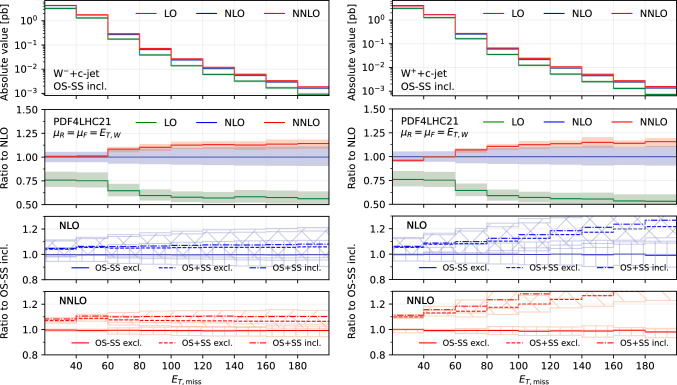
Comparison of predictions for the transverse missing energy $E_{T,\textrm{miss}}$, in the $W^-$+$c$-jet (left) and $W^+$+$c$-jet (right) processes. Panels from top to bottom: differential distribution at different orders; ratio of differential distributions to NLO; ratio of (OS−SS, excl.), (OS+SS, excl.) and (OS+SS, incl.) distributions to (OS−SS, incl.) at NLO; same for NNLO

**Fig. 5:**
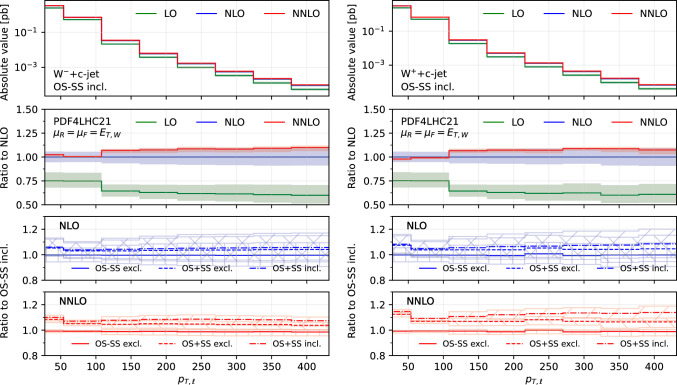
Comparison of predictions for the transverse momentum of the lepton $p_{T,\ell }$, in the $W^-$+$c$-jet (left) and $W^+$+$c$-jet (right) processes. Panels from top to bottom: differential distribution at different orders; ratio of differential distributions to NLO; ratio of (OS−SS, excl.), (OS+SS, excl.) and (OS+SS, incl.) distributions to (OS−SS, incl.) at NLO; same for NNLO

In order to study how predictions are affected by these requirements on the number and relative sign of $c$-jets, in some of the plots below we study variations of the setup. In particular, we will further consider the *exclusive* setup i.e. we require the presence of one and only one $c$-jet in each event (but we allow for any number of flavourless jets). We will also individually consider OS and SS events, and their sum OS+SS i.e. by not applying any OS−SS subtraction. In such cases, we will adopt the notation incl./excl. and OS−SS/OS+SS/OS/SS, to denote a specific setup. Where not indicated, we understand the default setup (OS−SS incl.).

Our results pass the usual checks routinely done in the context of a NNLOJET calculation (*spike-tests* [49] at real, real-virtual and double-real level; cancellation of infrared poles at virtual, real-virtual and double-virtual level; independence of the results from the technical cut at real, real-virtual and double-real level). The NNLO QCD corrections to $W+c$-jet production were computed previously in [13, 14]. These results were used in the recent CMS study of $W+c$-jet production [18] at 13 TeV. We cross checked our numbers for the fiducial cross section with Table 12 of [18], by performing dedicated computations for the CMS setup, finding good agreement at all perturbative orders, and for the OS/SS/OS−SS components separately.

### Fiducial cross sections

In this section, we present numbers for the fiducial cross section at different orders and for different setups. In Tables 1 and 2 we show results for the $W^+$+$c$-jet and $W^-$+$c$-jet processes respectively. Results are organised by perturbative order (rows) and setup (columns). Each row corresponds to the cross section at LO ($\sigma ^{\textrm{LO}}$), NLO ($\sigma ^\textrm{NLO}$) or NNLO ($\sigma ^{\textrm{NNLO}}$), or to the NLO ($\Delta \sigma ^{\textrm{NLO}}$) or NNLO ($\Delta \sigma ^{\textrm{NLO}}$) contribution to the total cross section. Each column corresponds to a particular setup, as explained in Sect. 3.1: OS−SS incl., OS−SS excl., OS+SS incl., OS+SS excl.. We further show the theory-uncertainty envelope associated to 7-point scale variation, expressed as percentage of the reported central value. The statistical Monte Carlo error on the calculation is indicated as an uncertainty on the last digit. In Table 3, we consider the ratio of fiducial cross sections for the $W^+$+$c$-jet and $W^-$+$c$-jet processes,

14 $$\begin{aligned} \mathcal {R}^\pm _c = \frac{\sigma (W^++c\text {-jet})}{\sigma (W^-+c\text {-jet})}. \end{aligned}$$

We show results for such a ratio at LO, NLO and NNLO (rows), in different setups (columns).

For both the individual processes $W^+$+$c$-jet and $W^-$+$c$-jet and for the ratio, we note excellent perturbative convergence, with small NNLO corrections and a converging pattern. The size of the theory uncertainty band progressively decreases when moving from LO to NNLO, with an uncertainty of ±10% at LO, $\pm 5$% at NLO and $\pm 1$–2% at NNLO for $W^\pm$+$c$-jet . As for $\mathcal {R}^\pm _c$, the decrease in size is even more pronounced, with an uncertainty of ±20% at LO, $\pm 10$% at NLO and $\pm 2$–3% at NNLO.

Moving to the comparison of different setups, we notice interesting hierarchies between the numbers in the tables. At LO, the fiducial cross section is always the same regardless of the setup, due to the presence of a single OS charm quark in the final state. When moving to NLO or NNLO, thus allowing for the presence of more charm quarks or anti-quarks in the event, the size of the difference between OS+SS and OS−SS increases, with a larger difference at NNLO than at NLO, and in $W^+$+$c$-jet than in $W^-$+$c$-jet. The difference between the inclusive and exclusive setup is more moderate, with numbers usually compatible within the scale variation uncertainties, and with a larger difference at NNLO than at NLO. The latter observation could be explained by the fact that the probability of having two or more $c$-jets in the event is small, where there are at most 2 and 3 charm (anti-)quarks in the event at NLO and at NNLO respectively. Similar comments apply to $\mathcal {R}^\pm _c$.

Finally, we note that the values of $\mathcal {R}^\pm _c$ in Table 3 are all smaller than 1, whatever the perturbative order and the setup i.e. the fiducial cross section for $W^+$+$c$-jet is always (slightly) smaller than the fiducial cross section for $W^-$+$c$-jet. This fact can be explained by an analysis of the couplings allowed by the CKM matrix and the behaviours of the parton distribution functions of the proton. At LO, the size of the contribution proportional to $|V_{cs}|$ is equivalent for $W^{+}+\bar{c}$ and $W^{-}+{c}$, because the strange and anti-strange PDFs are similar. However, the subleading contribution proportional to $|V_{ds}|$ is different between $W^{+}+\bar{c}$ and $W^{-}+{c}$: namely, the down PDF contributing to $W^{-}+{c}$ features a valence component, which is missing in the anti-down PDF contributing to $W^{+}+\bar{c}$. Hence, the cross section for $W^-$+$c$-jet is larger than for $W^+$+$c$-jet at LO, and higher-order corrections are not large enough to alter this simple picture. This insight will be instrumental in explaining differences in behaviour between the differential distributions for $W^+$+$c$-jet and $W^-$+$c$-jet shown in Sect. 3.3, and will be further explored in Sect. 4, where the contributions of individual partonic channels to the total cross section will be presented.

### Differential distributions

**Fig. 6:**
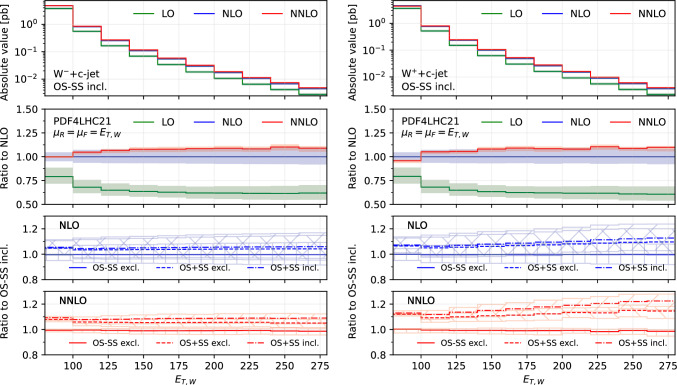
Comparison of predictions for the transverse energy $E_{T,W}$, in the $W^-$+$c$-jet (left) and $W^+$+$c$-jet (right) processes. Panels from top to bottom: differential distribution at different orders; ratio of differential distributions to NLO; ratio of (OS−SS, excl.), (OS+SS, excl.) and (OS+SS, incl.) distributions to (OS−SS, incl.) at NLO; same for NNLO

In this section we present differential distributions for several observables of interest, for both the $W^+$+$c$-jet and $W^-$+$c$-jet process. We consider the absolute rapidity of the lepton from the $W^{\pm }$ decay, $|y_\ell |$ (Fig. 1), the absolute pseudorapidity of the leading-$p_T$ $c$-jet, $|\eta _{j_c}|$ (Fig. 2), the transverse momentum of the leading-$p_T$ $c$-jet, $p_{T,j_c}$ (Fig. 3), the transverse missing energy, $E_{T,\textrm{miss}}$ (Fig. 4), the transverse momentum of the lepton from the $W^{\pm }$ decay, $p_{T,\ell }$ (Fig. 5) and the transverse energy $E_{T,W}$ defined as in (12) (Fig. 6).

Figures 1, 2, 3, 4, 5 and 6 are organised in the following way. On the left we show distributions for $W^-$+$c$-jet, on the right for $W^+$+$c$-jet. Each column has four panels, depicting: absolute value of the differential distribution at LO, NLO and NNLO in the OS−SS incl. setup (1st panel from the top); ratio of distributions in the OS−SS incl. setup at LO, NLO, NNLO to NLO prediction (2nd panel from the top); ratio of OS−SS excl., OS+SS excl. and OS+SS incl. distributions to OS−SS incl. distribution at NLO (3rd panel from the top) and at NNLO (4th panel from the top).

**Fig. 7:**
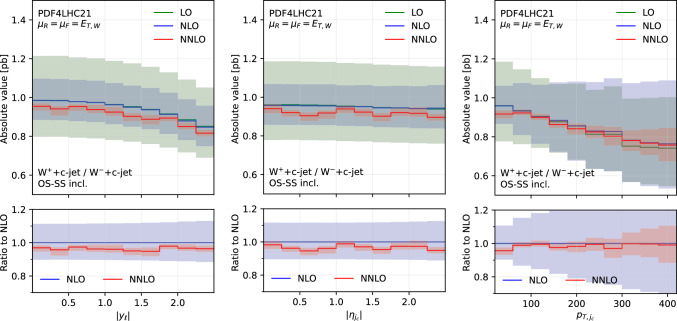
Comparison of differential distributions at different orders for the ratio $\sigma$($W^+$+$c$-jet)/$\sigma$($W^-$+$c$-jet), differential in $|y_\ell |$ (left), $|\eta _{j_c}|$ (middle), $p_{T,j_c}$ (right). The upper panels show the distributions in absolute value, whereas the lower panels show the ratio to the NLO prediction

Finally, in Fig. 7 we show the distributions differential in $|y_\ell |$ (first column), $|\eta _{j_c}|$ (second column) and $p_{T,j_c}$ (third column), by considering both distributions in absolute value at LO, NLO and NNLO (upper panels) and their ratio to the NLO prediction (lower panels). Here all the predictions are in the OS−SS incl. setup.

We first focus on the OS−SS incl. setup and we consider predictions at different perturbative orders. We observe in all of the Figs. 1, 2, 3, 4, 5,6 and 7 a nice perturbative convergence, with the NNLO curves contained within the NLO uncertainty bands, and with the NNLO uncertainty band always smaller by at least a factor of two compared to the NLO one. In the $p_{T,j_c}$, $E_{T,\textrm{miss}}$, $p_{T,\ell }$ and $E_{T,W}$ distributions, the NNLO curve lies just on the boundary of the NLO uncertainty band. For the ratio $\mathcal {R}^\pm _c$ in Fig. 7, we observe a drastic reduction of the theory uncertainty when moving from LO to NNLO for all the considered distributions, in line with what is observed for the ratio of fiducial cross sections in Sect. 3.2.

By focussing now on the comparison between different setups, we can draw similar conclusions to those already expressed in Sect. 3.2. Namely: the difference between excl. and incl. is greater at NNLO than at NLO (remember that at LO all the setups are the same); the difference between excl. and incl. is greater in the OS+SS case rather than in the OS−SS case; the difference between OS−SS and OS+SS is generally larger than the difference between excl. and incl. However, such differences are generally not flat in the differential distributions. While we observe that the differences between setups mildly depend on $|\eta _{j_c}|$, $p_{T,\ell }$ and $E_{T,W}$ for both $W^+$+$c$-jet and $W^-$+$c$-jet, we note a significant dependence on $|y_\ell |$, $p_{T,j_c}$ and $E_{T,\textrm{miss}}$. In particular, such a dependence is more pronounced at large values of $|y_\ell |$, $p_{T,j_c}$ and $E_{T,\textrm{miss}}$, and the behaviour of $W^-$+$c$-jet and $W^+$+$c$-jet is very different, with enhanced differences for $W^+$+$c$-jet between the different set-ups. We will return to this point in Sect. 4 below.

We conclude this Section by observing that the difference between excl. and incl. in the OS−SS case is very small in all the distributions, both at NLO and NNLO: it amounts to at most a couple of per-cent for high values of $p_{T,j_c}$. The OS−SS subtraction clearly helps in reducing the difference between the inclusive and exclusive prescription on the number of $c$-jets, because the events discarded when performing the OS−SS subtraction are a subset of events with more than one *c* parton in the event. However, it seems that OS−SS subtraction is very efficient in discarding events with more than one $c$-jet surviving the fiducial cuts. In other words, the inclusive two $c$-jets cross section is very small when applying the OS−SS subtraction.

## Partonic channel breakdown

In this section, we study how the individual partonic channels contribute to the total cross section. This analysis will be instrumental in understanding how higher-order radiative corrections in different setups affect the contributions coming from different PDFs.

**Fig. 8:**
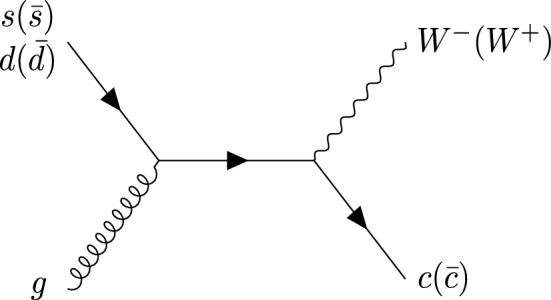
Born-level diagram for $W^+$+$c$-jet and $W^-$+$c$-jet

**Fig. 9:**
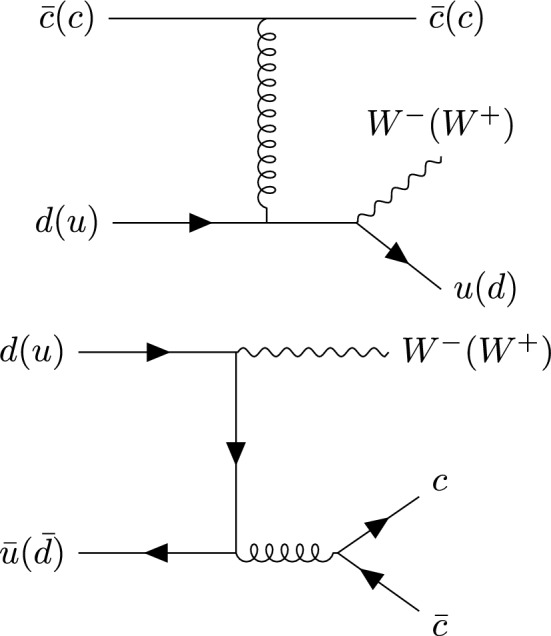
Example diagrams contributing to $W^+$+$c$-jet and $W^-$+$c$-jet from NLO onwards

We recall that at LO, $W+c$-jet production is mediated only through the Born-level process $sg \rightarrow W^-c$ and $\bar{s}g \rightarrow W^+\bar{c}$ (Fig. 8) and their CKM-suppressed *d*-quark initiated partner processes. They always result in OS final states. At higher orders, final states containing charm quarks can also be caused by a hard scattering process involving an initial-state charm quark or by the splitting of a final state gluon into a charm-anticharm pair, illustrated in Fig. 9.

In Tables 4 and 5 we present the contribution of each partonic channel in the $W^-$+$c$-jet and in the $W^+$+$c$-jet process, respectively. We provide numbers for OS at LO, NLO and NNLO, and for SS at NLO and NNLO (SS at LO is trivially zero). One can easily obtain the corresponding numbers for OS−SS and OS+SS. All the numbers refer to exclusive cross sections; the analogous numbers for inclusive cross sections are very similar, so throughout this section we will focus on the exclusive setup (which is more easily interpreted in terms of parton-level subprocesses), unless otherwise specified.

We have chosen to organise the partonic channels in the following way: we explicitly distinguish charm *c*($\bar{c}$) and strange *s*($\bar{s}$) (anti)quarks in the initial state, while denoting an (anti)quark of any other flavour as *q*($\bar{q}$). We do not differentiate between quarks and antiquarks i.e. we sum together contributions coming from quarks and antiquarks of the same flavour. In this way, we obtain 10 possible channels, as listed in the first column of Tables 4 and 5, whose contributions sum up to the total cross section.

**Table 4:** Breakdown of the fiducial cross section for $W^-$+$c$-jet in terms of the contributing partonic channels. We denote as $q ({\bar{q}})$ the quarks (antiquarks) of different flavour than $s ({\bar{s}})$ and $c ({\bar{c}})$. Furthermore, we do not distinguish between quarks and antiquarks e.g. the $c({\bar{c}})s({\bar{s}})$ row contains all the possible permutations of *c* and ${\bar{c}}$ with *s* and ${\bar{s}}$. All the numbers refer to exclusive cross sections

$W^-$+$c$-jet	OS LO	OS NLO	SS NLO	OS NNLO	SS NNLO
$c({\bar{c}})s({\bar{s}})$	0.0	$-0.1225(3)$	0.4852(2)	$-0.05(2)$	0.842(3)
$c({\bar{c}})c({\bar{c}})$	0.0	0.2158(1)	0.2062(2)	0.360(2)	0.351(1)
$c({\bar{c}})q({\bar{q}})$	0.0	1.2392(3)	1.3132(4)	1.958(4)	2.088(4)
$s({\bar{s}})q({\bar{q}})$	0.0	$-0.651(3)$	0.03134(1)	$-1.1(2)$	0.0537(2)
$s({\bar{s}})s({\bar{s}})$	0.0	$-0.2549(3)$	0.0	$-0.42(3)$	0.0
$q({\bar{q}})q({\bar{q}})$	0.0	1.0314(7)	0.9838(4)	1.73(2)	1.676(6)
$gq({\bar{q}})$	8.9255(6)	12.700(1)	0.0	12.7(2)	0.405(3)
$gs({\bar{s}})$	86.857(4)	123.002(8)	0.0	128.9(3)	$-0.0353(6)$
$$gc({\bar{c}})$$	0.0	0.0	0.0	$$-0.14 (2)$$	$$-0.057 (2) $$
*gg*	0.0	$-6.355(3)$	0.0	$-8.31 (1)$	0.0
Total	95.782(5)	130.806(1)	3.020(1)	135.6(5)	5.324 (9)

**Table 5:** Breakdown of the fiducial cross section for $W^+$+$c$-jet in terms of the contributing partonic channels. As in Table 4 we denote as $q ({\bar{q}})$ the quarks (antiquarks) of different flavour than $s ({\bar{s}})$ and $c (\bar{c})$. Furthermore, we do not distinguish between quarks and antiquarks e.g. the $c({\bar{c}})s({\bar{s}})$ row contains all the possible permutations of *c* and ${\bar{c}}$ with *s* and ${\bar{s}}$. All the numbers refer to exclusive cross sections

$W^+$+$c$-jet	OS LO	OS NLO	SS NLO	OS NNLO	SS NNLO
$$c({\bar{c}})s({\bar{s}})$$	0.0	$$-0.1191(9)$$	0.4752(4)	$$-0.13(2) $$	0.838(1)
$c({\bar{c}})c({\bar{c}})$	0.0	0.2151(3)	0.2047(3)	0.3316(5)	0.3246(6)
$c({\bar{c}})q({\bar{q}})$	0.0	1.948(3)	1.988(4)	2.945(6)	3.038(6)
$$s({\bar{s}})q({\bar{q}})$$	0.0	$$-0.649(9)$$	0.0673(1)	$$ -1.9(3)$$	0.1157(3)
$$s({\bar{s}})s({\bar{s}})$$	0.0	$$-0.258(1)$$	0.0	$$-0.55(5) $$	0.0
$q({\bar{q}})q({\bar{q}})$	0.0	1.431(2)	1.409(2)	2.35(2)	2.423(6)
$gq({\bar{q}})$	5.8299(7)	8.257(2)	0.0	10.1(4)	0.508(4)
$gs({\bar{s}})$	85.51(1)	121.04(3)	0.0	126.3(6)	$-0.0430(4)$
$gc({\bar{c}})$	0.0	0.0	0.0	0.02(2)	$-0.0293(7)$
*gg*	0.0	$$-6.34(1)$$	0.0	$$ -13.62(6)$$	0.0
Total	91.34(1)	125.51(4)	4.146(4)	125.9(7)	7.17(1)

**Fig. 10:**
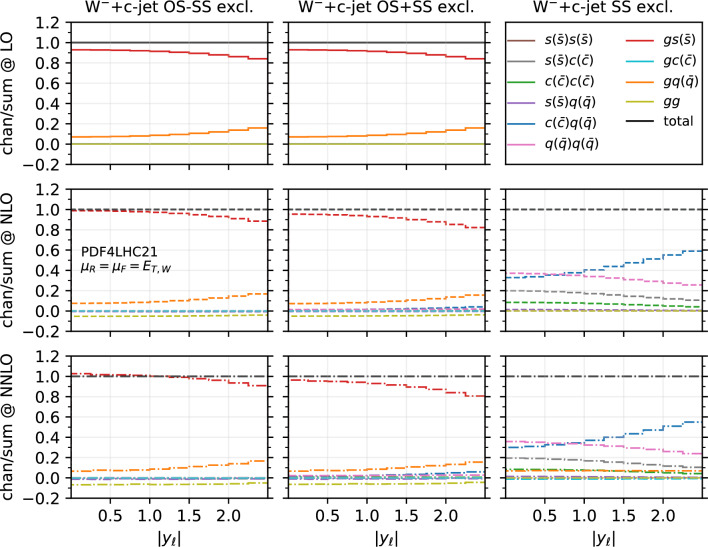
Fractional contribution of partonic channels to the total result at different perturbative orders, for the $W^-$+$c$-jet process, differential in $|y_{\ell }|$. The three columns correspond to different setups: OS−SS excl. (left), OS+SS excl. (middle), SS excl. (right). The three rows correspond to different perturbative orders: LO (top), NLO (middle), NNLO (bottom)

**Fig. 11:**
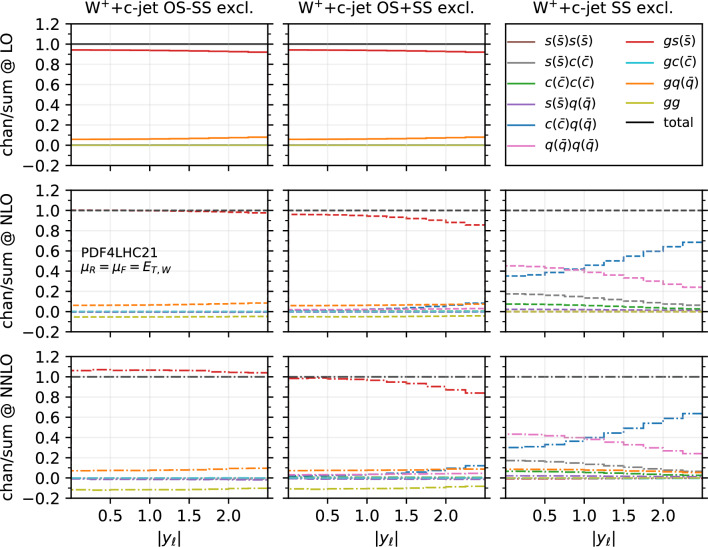
Fractional contribution of partonic channels to the total result at different perturbative orders, for the $W^+$+$c$-jet process, differential in $|y_{\ell }|$. The three columns correspond to different setups: OS−SS excl. (left), OS+SS excl. (middle), SS excl. (right). The three rows correspond to different perturbative orders: LO (top), NLO (middle), NNLO (bottom)

**Fig. 12:**
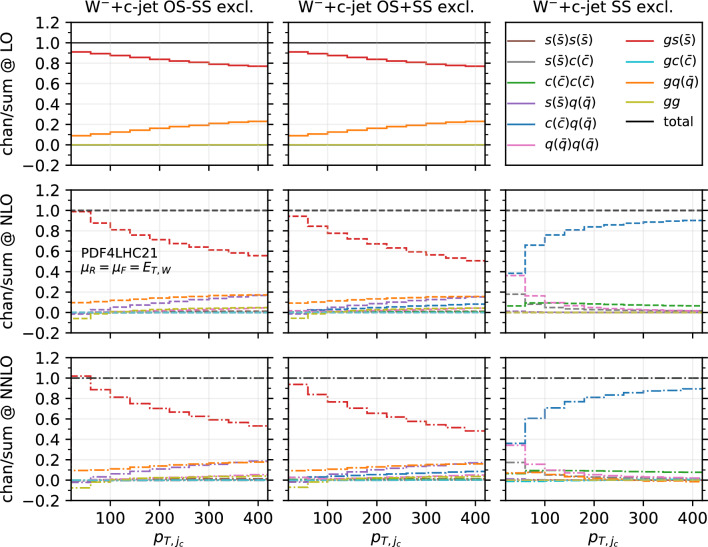
Fractional contribution of partonic channels to the total result at different perturbative orders, for the $W^-$+$c$-jet process, differential in $p_{T,j_c}$. The three columns correspond to different setups: OS−SS excl. (left), OS+SS excl. (middle), SS excl. (right). The three rows correspond to different perturbative orders: LO (top), NLO (middle), NNLO (bottom)

**Fig. 13:**
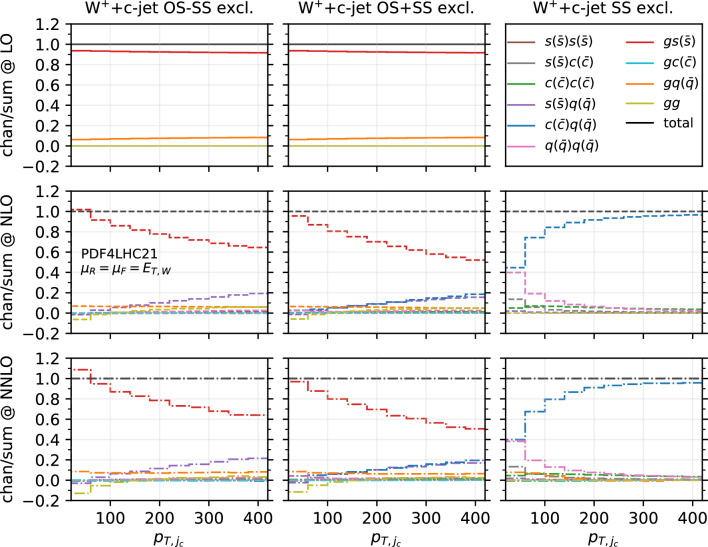
Fractional contribution of partonic channels to the total result at different perturbative orders, for the $W^+$+$c$-jet process, differential in $p_{T,j_c}$. The three columns correspond to different setups: OS−SS excl. (left), OS+SS excl. (middle), SS excl. (right). The three rows correspond to different perturbative orders: LO (top), NLO (middle), NNLO (bottom)

**Fig. 14:**
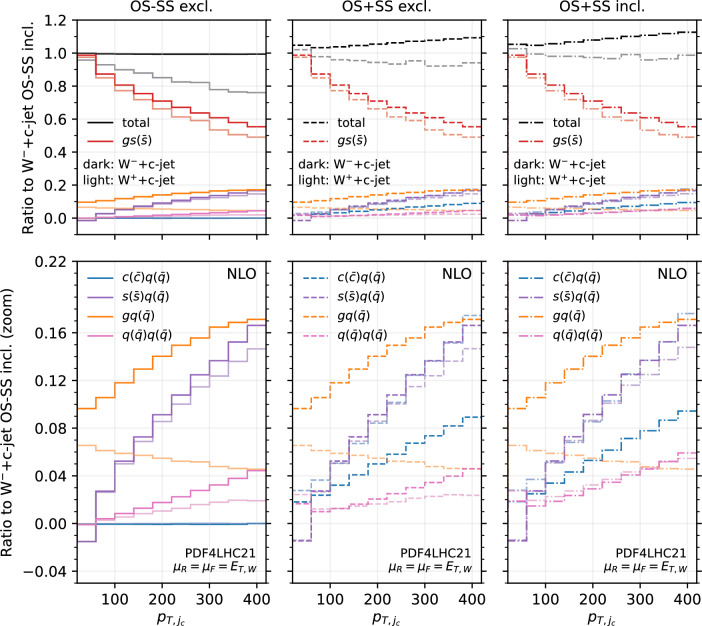
Analysis of the channels contributing to the $p_{T,j_c}$ distribution at NLO for OS−SS excl. (left), OS+SS excl. (middle) and OS+SS incl. (right). All the curves are normalised to the $W^-$+$c$-jet OS−SS incl. NLO distribution. The lower panel is just a zoom of the upper panel. Darker colours refer to $W^-$+$c$-jet, lighter colours refer to $W^+$+$c$-jet

**Fig. 15:**
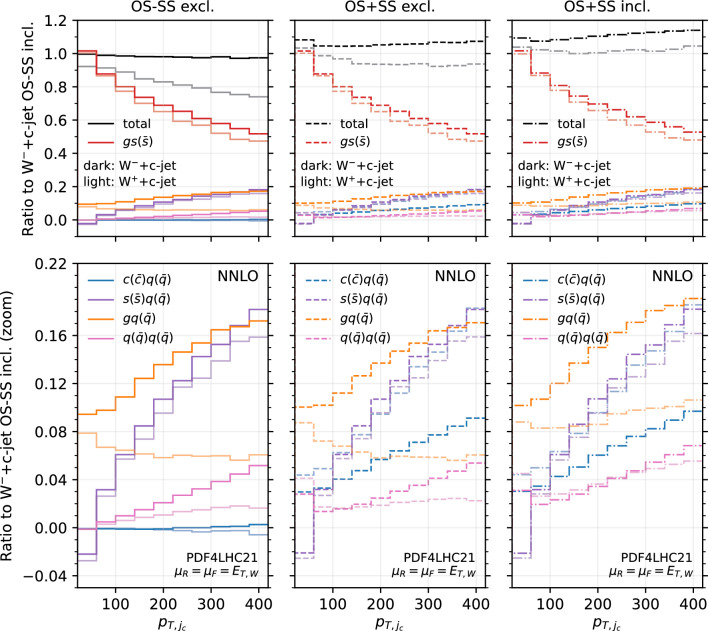
Analysis of the channels contributing to the $p_{T,j_c}$ distribution at NNLO for OS−SS excl. (left), OS+SS excl. (middle) and OS+SS incl. (right). All the curves are normalised to the $W^-$+$c$-jet OS−SS incl. NLO distribution. The lower panel is just a zoom of the upper panel. Darker colours refer to $W^-$+$c$-jet, lighter colours refer to $W^+$+$c$-jet

At all perturbative orders, the by far dominant contribution to the fiducial cross section in OS events comes from the $gs(_)$ channel, which amounts to 90% of the total. The second largest contribution (6–10%) to OS events comes from the $gq(\bar{q})$ channel. Such a contribution is slightly larger for $W^-$+$c$-jet: as already explained in Sect. 3.2, this is related to the presence of the *d* PDF in $W^-$+$c$-jet as opposed to the presence of $\bar{d}$ in $W^+$+$c$-jet. The third largest contribution (5–10%) comes from the *gg* channel, with a negative sign, partially compensating the $gq(\bar{q})$ contribution. In some cases, the *gg* channel can be even larger than the $gq(\bar{q})$ one (for instance in $W^+$+$c$-jet for OS at NNLO). All the other channels contribute much less to the total cross section (at most a few per-cent each).

It is interesting to compare the OS numbers for some channels with the analogous ones for SS. We notice that both at NLO and at NNLO, both for $W^+$+$c$-jet and for $W^-$+$c$-jet, the $c(c($ channel, the $c(q(\bar{q})$ channel and the $q(\bar{q})q(\bar{q})$ channel are numerically very similar between OS and SS. Hence when performing the OS−SS subtraction, we are enhancing the channels featuring a (anti)strange PDF, by removing channels with quarks of other flavours. The channels with a gluon PDF ($gq(\bar{q})$ and *gg*) still survive after the OS−SS subtraction.

In order to investigate how the overall picture is affected by different kinematical regions of phase space, we also investigate selected differential distributions. We focus on the $|y_\ell |$ and $p_{T,j_c}$ observables, and we consider the fractional contribution of each individual channel at each perturbative order for each bin of the corresponding differential distributions. The results are shown in Fig. 10 ($|y_\ell |$ in $W^-$+$c$-jet), Fig. 11 ($|y_\ell |$ in $W^+$+$c$-jet), Fig. 12 ($p_{T,j_c}$ in $W^-$+$c$-jet) and Fig. 13 ($p_{T,j_c}$ in $W^+$+$c$-jet). In each figure, we plot the contribution of each partonic channel normalised to the total at LO (1st row from the top), total NLO (2nd row from the top) and total NNLO (3rd row from the top). The left and the middle columns are in the OS−SS excl. and OS+SS excl. setups, respectively, whereas the right column is in the SS setup. We chose to plot OS−SS excl. and OS+SS excl.  in order to have a complementary information to the one provided in Tables 4 and 5. Instead, in the SS column, one can better appreciate the difference between the several curves, given that the dominant $gs(_)$ component is absent. The channel decompositions in Figs. 10, 11, 13 are obtained at a central scale $\mu _R = \mu _F = E_{T,W}$. They are only mildly dependent in the choice of central scale, varying by at most 5% under factor two variations around the central scale, mainly in the form of shifts between the $gs({\bar{s}})$ and *gg* channels.

We first focus on the OS−SS excl. and OS+SS excl. setups. In all plots, we notice the dominance of the $gs(_)$ channel, as already observed for the fiducial cross sections. However, it can be seen that for large values of $|y_\ell |$ and $p_{T,j_c}$, the fractional contribution of $gs(_)$ decreases, with the other channels starting to contribute more. In particular, in the $|y_\ell |$ distribution, we observe that $gs(_)$ is always very close to 1 for most of the rapidity range, except for $|y_\ell | \gtrsim 2.0$ where it decreases to 0.8. The overall picture is only mildly affected by the perturbative order. In contrast, in the $p_{T,j_c}$ distribution, we note a sharp decrease of the $gs(_)$ contribution as $p_{T,j_c}$ increases: while at LO $gs(_)$ is around 0.9 for low-$p_{T,j_c}$ values down to 0.8 for high-$p_{T,j_c}$ values, at NLO and NNLO it goes down to 0.5–0.6 for $p_{T,j_c} \sim 400$ GeV. Other channels then give a non-negligible contribution at high transverse momenta: the $gq(\bar{q})$ and $s(_)q(\bar{q})$ channels both in the OS−SS and OS+SS setup; the $c(q(\bar{q})$ channels only in the OS+SS setup. Indeed, by comparing left columns (OS−SS) with the middle columns (OS+SS), the effect of the OS−SS subtraction is evident, with the curve associated to $c(q(\bar{q})$ close to zero on the left. As for the *gg* channel, its contribution mildly depends on $|y_\ell |$, being negative and constant in the whole rapidity range. Instead, it peaks at low-$p_{T,j_c}$ at NLO and NNLO (where the total cross section is larger), with a negligible contribution at large transverse momenta.

It is also interesting to note how the individual channels behave between $W^-$+$c$-jet and $W^+$+$c$-jet. For instance, already at LO, the behaviour of the $gq(\bar{q})$ channel both at large rapidities and at large transverse momenta is different, with a larger contribution of $gq(\bar{q})$ in $W^-$+$c$-jet. These kinematic regions mainly receive contributions from PDFs at large momentum fraction; hence, the plots confirm that the origin of the difference between $W^-$+$c$-jet and $W^+$+$c$-jet to be related to the valence component of the *d* PDF, which is absent for the $\bar{d}$ PDF. Equally noteworthy is the difference in size between the $c(q(\bar{q})$ and the $q(\bar{q})q(\bar{q})$ channels in $W^+$+$c$-jet and $W^-$+$c$-jet at NLO and NNLO in the OS+SS excl. setup.

We now consider the SS plots i.e. the column on the right in Figs. 10, 11, and 13. We see that both $c(q(\bar{q})$ and $q(\bar{q})q(\bar{q})$ are equally dominant for small rapidity values, with $c(q(\bar{q})$ becoming larger and $q(\bar{q})q(\bar{q})$ becoming smaller at large rapidities, both at NLO and NNLO. The situation is similar in the $p_{T,j_c}$ distribution, but starting from $p_{T,j_c} \gtrsim 300$ GeV the $c(q(\bar{q})$ channel constitutes the totality of the SS cross section, with the $q(\bar{q})q(\bar{q})$ near to zero. It is likely that in these events at large-$p_{T,j_c}$ the SS *c*-parton comes directly from the PDFs: if it were radiatively generated, then other channels would also contribute.

Having scrutinized in detail how contributions to the cross sections are distributed among the various channels, we now return to consider the bottom panels of Fig. 3. Namely, understanding why the behaviour of the considered setups is so different between $W^-$+$c$-jet and $W^+$+$c$-jet in the $p_{T,j_c}$ distribution. This will give us the opportunity to further investigate the correlation between PDFs and cross sections for $W^-$+$c$-jet and $W^+$+$c$-jet.

Towards this aim, we consider again the $p_{T,j_c}$ distribution at NLO (Fig. 14) and at NNLO (Fig. 15). However we now include curves with the contributions of the most sizeable channels, and superimpose $W^+$+$c$-jet and $W^-$+$c$-jet on the same plot, by choosing as common normalisation factor the $W^-$+$c$-jet OS−SS incl. distribution. In this way, we can determine the relative size of contributions between $W^+$+$c$-jet and $W^-$+$c$-jet. The darker colours refer to $W^-$+$c$-jet, whereas the lighter ones to $W^+$+$c$-jet. We show results for the OS−SS excl. setup (left frames), the OS+SS excl. setup (middle frames), the OS+SS incl. setup (right frames). The black curves in the upper left plots of Figs. 14 and 15 coincide with the blue (NLO) and red (NNLO) curves in the left plot in Fig. 3. Likewise, the gray curves in the upper left plots of Figs. 14 and 15 correspond to the blue and red curves in the right plot in Fig. 3, but they do not coincide as they have a different normalisation.

We observe several important features. At NLO for both $W^-$+$c$-jet and $W^+$+$c$-jet, the difference between OS+SS excl. and OS−SS excl. is driven by the $c(q(\bar{q})$ channel, and the difference between OS+SS excl. and OS+SS incl. is driven by $q(\bar{q})q(\bar{q})$. At NNLO, similar observations hold, with $gq(\bar{q})$ channel responsible for further increasing the difference between OS+SS excl. and OS+SS incl. Hence, explaining the lower panels of Fig. 3 amounts to understanding why the $c(q(\bar{q})$, $q(\bar{q})q(\bar{q})$ and $gq(\bar{q})$ channels are so different in size between $W^-$+$c$-jet and $W^+$+$c$-jet.

Starting from the $c(q(\bar{q})$ channel, from the discussion above we know that in the high-$p_{T,j_c}$ region these events feature a SS *c*-parton coming directly from the PDFs. Therefore, the quark line coupling to the *W*-boson is unconstrained in terms of flavour. A typical diagram of such a configuration is displayed in Fig. 9 on the left. The largest contribution in the large-*x* region comes from the *d* valence PDF in the case of $W^{-}$ and from the *u* valence PDF in the case of $W^{+}$. The latter is approximately twice of the former, hence the factor of roughly 2 between the contribution of the $c(q(\bar{q})$ channel in OS+SS for $W^-$+$c$-jet and for $W^+$+$c$-jet in the large-$p_{T,j_c}$ region is easily explained.

One can explain in a similar manner why the $q(\bar{q})q(\bar{q})$ and $gq(\bar{q})$ channels induce the difference between the incl. and the excl. setup, and why such a difference is greater for $W^+$+$c$-jet compared to $W^-$+$c$-jet. A typical diagram for $q(\bar{q})q(\bar{q})$ is shown in Fig. 9 on the right. In this case, the charm is generated radiatively, hence we are summing over the flavour combinations of the two incoming quarks. Again the largest contributions in the large-*x* region comes from the $u\bar{d}$ channel in $W^+$+$c$-jet and from the $d\bar{u}$ channel in $W^-$+$c$-jet, so one recover the factor of 2 in difference. Similar considerations apply to the $gq(\bar{q})$ channels, which however features a secondary pair of charm quarks only starting from NNLO.

We conclude this section by noting that the findings on the relative importance of different partonic channels are very robust under changes of the PDF set. By comparing our default PDF4LHC21 predictions with results obtained using the ABMP16 [4] or NNPDF4.0 [7] PDF sets, we observe that the $W^+$+$c$-jet cross sections change by $\pm 2\%$ and the $W^-$+$c$-jet cross sections by $\pm 5\%$. The variation is uniform in the rapidity distributions and increases towards large transverse momenta. It is of comparable size in the OS+SS and OS−SS combinations. The relative decomposition of these cross sections into different partonic subprocesses remains largely unchanged under a variation of the PDF sets.

## Conclusions

In this paper, we presented a new calculation of *W*+charm-jet production up to NNLO in QCD. We employed a new flavour-dressing procedure [23] to define charm-jets in an IRC safe manner. Our results confirm an earlier calculation [13, 14], applied to the kinematics of a recent CMS measurement [18]. A detailed decomposition into different partonic channels demonstrated that the predominant contribution from initial states containing strange quarks is maintained in most kinematical distributions even when higher-order corrections are included. The efficiency of the OS−SS subtraction in removing contributions from secondary charm production is clearly demonstrated by the channel decomposition. This decomposition also explains the consistently larger magnitude of the $W^-$+$c$-jet over $W^+$+$c$-jet cross sections to be due to contributions from CKM-suppressed *d*-valence quark initiated processes.

Our results demonstrate the practical application of flavour dressing [23] in NNLO QCD predictions. They will enable the usage of *W*+charm-jet production observables in future global NNLO PDF fits and thus enable a precise flavour composition of the quark content of the nucleon.

## Data Availability

This manuscript has no associated data or the data will not be deposited. [Authors’ comment: For this publication only computer-generated pseudo data were used. These have been obtained with a private Monte Carlo integrator.]
